# Perioperative Considerations in Alpha-Gal Syndrome: A Review

**DOI:** 10.7759/cureus.53208

**Published:** 2024-01-30

**Authors:** John Leder, Anna Diederich, Bhavik Patel, Mark Bowie, Christian M Renwick, Venkat Mangunta

**Affiliations:** 1 Department of Cardiothoracic Intensive Care Nursing, University of Virginia, Charlottesville, USA; 2 Department of Anesthesiology, University of Virginia School of Medicine, Charlottesville, USA; 3 Department of Clinical Pharmacy, University of Virginia School of Medicine, Charlottesville, USA; 4 Division of Cardiothoracic Anesthesia, Department of Anesthesiology, Lehigh Valley Health Network, Allentown, USA

**Keywords:** pharmacology, pre-operative evaluation, allergy, alpha gal syndrome, critical care, anesthesiology

## Abstract

Galactose-⍺-1, 3-galactose (alpha-gal) is an oligosaccharide found in mammalian tissues that causes allergic reactions in patients with alpha-gal syndrome (AGS). AGS is a hypersensitivity reaction notable for both immediate and delayed allergic and anaphylactic symptoms. As a tick-based disease, AGS has gained increasing prevalence across the United States and can have a significant influence on which medications are safe for patients. Many medications used within the operating room and intensive care units have inactive ingredients that can be mammalian-derived and therefore should be vetted before administering to patients with AGS. Management of patients with AGS involves diligent action in the preoperative and perioperative settings to reduce patient exposure to potentially harmful medications. In conducting a comprehensive risk stratification assessment, the anesthesia team should identify any at-risk patients and determine which medications they have safely tolerated in the past. Despite obtaining a complete history, not all patients with AGS will be identified preoperatively. The perioperative team should understand which common medications pose a risk of containing alpha-gal moieties (e.g., heparins, gelatin capsules, vaccines, lidocaine patches, surgifoam, etc.​​). For this reason, this paper includes a compendium of common anesthetic medications that have been cross-referenced for ingredients that have the potential to cause an AGS reaction. Any potentially unsafe medications have been identified such that medical providers can cross-reference with the ingredients listed at their respective institutions.

## Introduction and background

Galactose-⍺-1, 3-galactose (alpha-gal) is an oligosaccharide found in most mammalian tissues, excluding old-world monkeys and apes [1]. Hypersensitivity reactions to cetuximab led Dr. Thomas Platts-Mills and his team at the University of Virginia to first identify the alpha-gal molecule as causing an immunoglobulin E (IgE)-mediated immune reaction in 2008 [2]. Typically, these IgE-mediated reactions to alpha-gal may present in one of two ways. The first is a delayed response occurring approximately 2-6 hours after oral ingestion of certain products (i.e., “red meats”, gelatin-coated medications, animal-based glycerin, animal-derived magnesium stearate, bovine extract, etc.) [3], with typical hypersensitivity type I symptoms such as gastrointestinal distress, urticaria, and anaphylaxis. Conversely, parenteral introduction (such as IV medications) can cause an immediate anaphylactic presentation [3-4]. However, reactions may be highly varied and can include non-classical symptoms such as abdominal pain without the presence of hives, oropharyngeal edema, or pruritus [5]. Significant intraindividual variability has been reported wherein the degree of a reaction is not constant across exposures. One proposed explanation may relate to the form of the alpha-gal moiety itself and/or the presence of cofactors, such as non-steroidal anti-inflammatory medications (NSAIDs), alcohol, or exercise [3]. This intraindividual variability also extends to an individual’s sensitivity to various triggers, likely influenced by the quantity of alpha-gal in different products [6]. For example, one patient may react to microgram quantities of alpha-gal in milk, while another may experience reactions to any quantity of gelatin [7], and a third may experience reaction only after consuming large amounts of pork [3].

The geographic distribution of “alpha-gal syndrome” (AGS) or “red meat allergy” has a high incidence in the southeastern United States [1,8], coinciding with the endemic lone star tick, *Amblyomma americanum* [9]. However, the geographic range of *Amblyomma americanum* may expand northward as indicated by increasing case reports of AGS in Minnesota [10]. Conversely, fewer AGS cases have been reported in the Gulf Coast and Texas [9,11]. These shifts are further supported by dynamic changes in climate and migration patterns of the lone star tick’s host organism, that is, the white-tailed deer (Odocoileus virginianus) [9]. Furthermore, AGS has been reported worldwide with various other triggering species indicted [12], altogether indicating the expanding importance of an awareness of AGS and its clinical ramifications

With this review, the authors aim to provide a basic overview of how alpha-gal can impact anesthetic care along with a comprehensive safety profile of medications common to the perioperative and critical care settings for patients with AGS. As such, medications were identified if they possess inactive ingredients capable of inducing a hypersensitivity reaction in patients with AGS.

## Review

Alpha-gal in medications

The alpha-gal epitopes that trigger an immune response are not produced naturally in humans [13-14]. However, following exposure to certain tick bites, innate anti-Gal-producing B cells can undergo isotype switching to produce anti-Gal IgE. These newly formed anti-Gal IgE molecules can then bind to alpha-gal, including that found in common perioperative medications, and induce a hypersensitivity reaction [15-16]. These anti-Gal immunoglobulins are the most abundant naturally occurring antibodies (accounting for roughly 1% of immunoglobulins). Non-B blood groups have a greater number of anti-Gal antibodies and therefore are at an increased risk of having immunological pathogenesis [13].

Compounds used in pharmaceuticals that are derived from mammalian origins have the potential to contain alpha-gal due to their source. Veiled ingredients that may be mammalian derived, and therefore contain the alpha-gal moiety, include the following [17-20]:

· Arachidonic acid

· Arachidyl propionate

· Gelatin

· Glycerin

· Glycerol

· Heparin

· Lactic acid

· Lactose monohydrate

· Lanolin

· Magnesium stearate

· Milk proteins

· Monoclonal antibodies

· Myristic acid

· Oleic acid

· Stearate

· Stearic acid

· Thrombin

The source of the ingredient (animal versus plant) must be verified with the manufacturer as most medication monographs do not include this information. Some manufacturers do not report the source because they do not know the full derivation of all substances used in their medications [1]. Several of these ingredients (e.g., glycerin, oleic acid, stearic acid, gelatin, glycerol, magnesium stearate) may have non-animal-derived alternatives, which can be difficult to clearly delineate [1,19].

Meanwhile, the amount of alpha-gal in animal-derived ingredients has not been established. This lack of quantifiability, combined with intraindividual variability of symptom manifestation, means that clinicians must take special precautions in situations that have an increased probability of causing a systemic reaction. One such notable example of this is during heparin infusion with cardiopulmonary bypass. A retrospective review of 8,819 patients undergoing cardiac surgery at a single institution between 2007 and 2019 who received high-dose heparin identified 49 patients who were incidentally tested for alpha-gal, of whom 17 tested positive preoperatively [21]. A subanalysis was conducted for eight of the patients who had alpha-gal-specific IgE titers collected within 90 days prior to or after operation [22]. Of the 17 AGS-positive patients, four (24%) responded with a severe allergic reaction during cardiac surgery. Conversely, of the eight patients in the subanalysis, four (50%) responded with an allergic reaction. Median alpha-gal IgE serum titers were significantly higher in those who suffered an allergic reaction compared to those who did not. This difference in titers was statistically more pronounced in the subanalysis group, demonstrating that alpha-gal IgE titer is a critical factor in determining patients at a higher risk of developing a reaction [21]. These titers, however, are not static over time, and therefore it has been recommended to collect a titer as part of a comprehensive preoperative plan [21].

While further studies are necessary to assess the efficacy of premedication with steroids and antihistamines in patients with high alpha-gal titers, the risk-benefit ratio of administering steroids and antihistamines before receiving high-dose heparin favors this intervention to possibly mitigate adverse outcomes [21]. For reactions in which patients were not prophylactically given steroids and/or antihistamines, Hawkins et al. observed that patients were successfully managed with a combination of antihistamine, glucocorticosteroid, and vasopressors, with symptoms of an AGS reaction not persisting beyond 24 hours postoperatively [21].

Anesthetic implications

Management of patients with AGS involves diligent action in the preoperative and perioperative settings to reduce patient exposure to potentially harmful medications. In conducting a comprehensive risk stratification assessment, the anesthesia team should identify any at-risk patients and identify them as such in the electronic medical record. With AGS often difficult to detect, as well as requiring laboratory tests for confirmation, exploring pertinent risk factors outside of a basic drug allergy screen can prove crucial. Pertinent risk factors to consider include the following [1,2,9,13,19,23]:

· Living in the Eastern United States

· Occupational exposure to ticks and/or wildlife

· History of tick bites (or treatment for tick-bite-related diseases)

· Sensitivity to red meats (may wax and wane)

· Frequent gastrointestinal symptoms such as nausea, vomiting, and diarrhea (with or without cutaneous, respiratory, or cardiovascular impacts)

· Prior sensitivity to cetuximab

· Non-B blood groups

Patients with AGS may often have misdiagnosed or underreported symptoms such as gastrointestinal distress that may be attributed to irritable bowel syndrome or late-night symptoms (such as pruritus) requiring the regular use of antihistamines. Further complicating the situation, however, is that clinical symptoms of AGS may only arise in some patients after co-administration of factors such as alcohol or activity [19]. To properly diagnose AGS, clinicians must screen for common characteristics of AGS while clarifying potential sensitivities and severities of reaction on a person-by-person basis. More than 90% of AGS cases can be accurately diagnosed through a history of delayed reaction to red meat in combination with an IgE to alpha-gal serum >0.1 IU/mL [19].

While not necessary prior to proceeding with surgery, patients who screen positive for AGS should ideally be referred to an allergist for further evaluation preoperatively. Sensitivity to alpha-gal is measured with serum alpha-gal IgE levels, and patients with AGS should be regularly evaluated by an allergist, which includes monitoring trends in these serum immunoglobulin levels. It is important to note that even among patients with similar alpha-gal IgE serum levels, not all patients will have reactions to every ingredient containing alpha-gal, and serum level does not always correspond to the degree of reaction [24-25]. Therefore, in patients with a known history of AGS, a key component of their pre-anesthetic evaluation will be to determine which medications they have safely tolerated in the past. This can include everything ranging from over-the-counter medications for pain (such as acetaminophen and NSAIDs), ambulatory prescriptions, to induction agents during any prior surgeries.

Even with adequate screening, the perioperative team should understand which common medications pose a risk of containing alpha-gal moieties (e.g., heparins, gelatin capsules, vaccines, lidocaine patches, surgifoam, acetaminophen tablets, Hospira’s intravenous hydromorphone, and diphenhydramine tablets) [16,19,26]. Additionally, the anesthesiologist needs to understand the clinical manifestations of an alpha-gal hypersensitivity reaction and how to treat these symptoms. Medications used in the treatment of AGS-related hypersensitivity reactions mirror those used to treat other allergic/anaphylactic reactions that can occur perioperatively, such as antihistamines (H1 and H2 receptor antagonists), albuterol, epinephrine, corticosteroids, and IV fluid administration [1]. While the goal with any hypersensitivity reaction is to identify and discontinue the offending agent immediately, this may not always be possible with AGS, especially in the setting of a potentially delayed reaction.

Review methods

The authors compiled a list of medications commonly used by anesthesiologists within the operating room and intensive care unit, two situations in which anesthesiologists are likely to care for patients with AGS. Once a list of relevant medications was developed, each medication was reviewed in the National Library of Medicine’s DailyMed database (dailymed.nlm.nih.gov) [27]. Drug label info was downloaded, and all active and inactive ingredients were screened for compounds with potential alpha-gal cross-reactivity (listed above). Only single-medication drugs were included. For example, “aspirin” was included within the compendium, but “aspirin-caffeine” was not. This same exclusion was applied to medications included within procedural kits, in particular lidocaine. See Tables 1-8 for the complete list of evaluated medications.

**Table 1:** Intravenous, volatile, and local anesthetics This table details the most common intravenous, volatile, and local anesthetics used within the operating room. All volatile anesthetics and local anesthetics included in this review are free from any inactive ingredients that may potentiate an allergic reaction in patients with AGS. AGS, alpha-gal syndrome

Drug	Formulation	Safety profile	Potentially concerning ingredient(s)
Intravenous anesthetics			
Dexmedetomidine	Intravenous	Safe	-
Etomidate	Intravenous	Safe	-
Ketamine	Intravenous	Safe	-
Methohexital	Intravenous	Safe	-
Propofol	Intravenous	Potentially unsafe	Glycerol
Volatile anesthetics			
Desflurane	Inhalation	Safe	-
Isoflurane	Inhalation	Safe	-
Nitrous oxide	Inhalation	Safe	-
Sevoflurane	Inhalation	Safe	-
Local anesthetics			
Lidocaine	Intravenous	Safe	-
Lidocaine with epinephrine	Perineural	Safe	-

**Table 2:** Neuromuscular blocking drugs This table details the most common neuromuscular blocking drugs used within the operating room and the intensive care unit. All medications reviewed within this category are free from any inactive ingredients that may potentiate an allergic reaction in patients with AGS. AGS, alpha-gal syndrome

Drug	Formulation	Safety profile	Potentially concerning ingredient(s)
Cisatracurium	Intravenous	Safe	-
Rocuronium	Intravenous	Safe	-
Succinylcholine	Intravenous	Safe	-
Vecuronium	Intravenous	Safe	-

**Table 3:** Opioid and non-opioid analgesics This table details the most common opioid and non-opioid analgesics used within the operating room, the perioperative setting, and the intensive care unit. Except for hydromorphone, all intravenous analgesics included in this review are free from any inactive ingredients that may potentiate an allergic reaction in patients with AGS. Specific care should be taken with the oral analgesics evaluated as they contain inactive ingredients that may potentiate an allergic reaction. Drugs marked with an asterisk (*) have specific formulations from manufacturers that may be free from potential AGS reactants. See the supplemental data sheet for NDCs (details in the Acknowledgments).

Drug name	Formulation	Safety profile	Potentially concerning ingredient(s)
Opioid analgesics			
Fentanyl	Intravenous	Safe	-
Hydromorphone	Oral	Potentially unsafe	Glycerin, lactose (anhydrous, hydrous, and monohydrate), magnesium stearate, stearic acid
Hydromorphone	Intravenous	Potentially unsafe	Lactic acid (unspecified form), sodium lactate
Hydromorphone*	Intravenous	Safe	-
Remifentanil	Intravenous	Safe	-
Sufentanil	Intravenous	Safe	-
Non-opioid analgesics			
Acetaminophen	Oral (tablet)	Potentially unsafe	Gelatin, glycerin, lactose monohydrate, magnesium stearate, stearic acid
Acetaminophen*	Oral (capsule)	Potentially unsafe	Gelatin, glycerin, lactose monohydrate, oleic acid, stearic acid
Acetaminophen	Intravenous	Safe	-
Ketorolac	Intravenous	Safe	-

**Table 4:** Vasoactive agents This table details the most common vasoactive medications used within the operating room and the intensive care unit. Drugs marked with an asterisk (*) have specific formulations from specific manufacturers that may be free from potential AGS reactants. See the supplemental data sheet for NDCs (details in the Acknowledgments). AGS, alpha-gal syndrome

Drug	Formulation	Safety profile	Potentially concerning ingredient(s)
Clevidipine	Intravenous	Potentially unsafe	Glycerin, oleic acid
Ephedrine	Intravenous	Safe	-
Epinephrine	Intravenous	Safe	-
Esmolol	Intravenous	Safe	-
Labetalol	Intravenous	Safe	-
Metoprolol	Intravenous	Safe	-
Milrinone	Intravenous	Potentially unsafe	Lactic acid
Nitroglycerin	Intravenous	Safe	-
Norepinephrine	Intravenous	Safe	-
Phenylephrine	Intravenous	Safe	-
Vasopressin*	Intravenous	Potentially unsafe	Lactic acid

**Table 5:** Cholinergic drugs and antiemetics This table details the most common cholinergic medications and antiemetics used in the perioperative setting, the operating room, and the intensive care unit. All medications reviewed within these categories are free from any inactive ingredients that may potentiate an allergic reaction in patients with AGS. AGS, alpha-gal syndrome

Drug	Formulation	Safety profile	Potentially concerning ingredient(s)
Cholinergic drugs			
Atropine	Intravenous	Safe	-
Glycopyrrolate	Intravenous	Safe	-
Neostigmine	Intravenous	Safe	-
Antiemetics			
Dexamethasone	Intravenous	Safe	-
Ondansetron	Intravenous	Safe	-

**Table 6:** Antibiotics This table details the most common antibiotics used within the operating room, the perioperative setting, and the intensive care unit. All antibiotics included in this review are free from any inactive ingredients that may potentiate an allergic reaction in patients with AGS. AGS, alpha-gal syndrome

Drug	Formulation	Safety profile	Potentially concerning ingredient(s)
Ampicillin-sulbactam	Intravenous	Safe	-
Cefazolin	Intravenous	Safe	-
Cefoxitin	Intravenous	Safe	-
Metronidazole	Intravenous	Safe	-
Piperacillin-tazobactam	Intravenous	Safe	-
Vancomycin	Intravenous	Safe	-

**Table 7:** Anticoagulants and antiplatelets This table details the most common anticoagulants and antiplatelets used within the operating room, the perioperative setting, and the intensive care unit. Many of the anticoagulant and antiplatelet medications included in this review have inactive ingredients that could potentiate an allergic reaction in patients with AGS. If a patient has not tolerated the specific medication previously and alternative medications are available, then such alternatives should be preferentially used. Drugs marked with an asterisk (*) have specific formulations from specific manufacturers that may be free from potential AGS reactants. See the supplemental data sheet for NDCs (details in the Acknowledgments). AGS, alpha-gal syndrome

Drug	Formulation	Safety profile	Potentially concerning ingredient(s)
Anticoagulants			
Apixaban	Oral	Potentially unsafe	Anhydrous lactose, magnesium stearate
Argatroban	Intravenous	Safe	-
Bivalirudin	Intravenous	Safe	-
Enoxaparin	Intravenous, subcutaneous	Potentially unsafe	Potential porcine derivative
Heparin	Intravenous, subcutaneous	Potentially unsafe	Potential porcine derivative
Rivaroxaban	Oral	Potentially unsafe	Lactose monohydrate
Warfarin	Oral	Potentially unsafe	Lactose monohydrate, magnesium stearate
Antiplatelets			
Aspirin	Oral	Potentially unsafe	Magnesium stearate
Aspirin	Rectal	Safe	-
Clopidogrel*	Oral	Potentially unsafe	Lactose monohydrate
Prasugrel*	Oral	Potentially unsafe	Lactose monohydrate
Ticagrelor	Oral	Potentially unsafe	Magnesium stearate

**Table 8:** Miscellaneous medications This table details various miscellaneous medications used within the operating room, the perioperative setting, and the intensive care unit. Except for epoprostenol, all the other medications of the indicated formulations within this category are free from any inactive ingredients that may potentiate an allergic reaction in patients with AGS. The safety profile of medications such as diphenhydramine does not necessarily extend to oral formulations. AGS, alpha-gal syndrome

Drug	Formulation	Safety profile	Potentially concerning ingredient(s)
Aminocaproic acid	Intravenous	Safe	-
Calcium chloride	Intravenous	Safe	-
Calcium gluconate	Intravenous	Safe	-
Diphenhydramine	Intravenous	Safe	-
Epoprostenol	Inhalation	Potentially unsafe	Metabolite of arachidonic acid
Magnesium sulfate	Intravenous	Safe	-
Prothrombin complex concentrate	Intravenous	Safe	-
Sugammadex	Intravenous	Safe	-
Tranexamic acid	Intravenous	Safe	-

Many of the medications in this list are produced by multiple manufacturers within the United States and internationally. Those medications listed as “Safe” indicate that all formulation national-drug codes (NDCs) in the database have been reviewed and do not contain ingredients with AGS reaction potential. Conversely, medications listed as “Potentially Unsafe” indicate that one or more of the manufacturers for the respective medications use these concerning ingredients. Any potentially unsafe medications have been identified such that medical providers should cross-reference with the ingredients listed at their respective institutions. This list is meant to complement any lists established and maintained by local hospital-based pharmacies as formulations are often updated per patenting and distribution regulations. Additionally, different lots by the same manufacturer, even with the same inactive ingredients, may not necessarily have the same concentrations of alpha-gal [1]. Considering the potential for patient morbidity and mortality, specific care and consideration should be exercised before administering any medication to a patient with AGS, especially if prior exposure to the medication is unknown.

## Conclusions

AGS remains a relatively underdiagnosed disorder with significant implications for anesthetic care, including delayed anaphylactic reactions. As the identification of AGS continues to increase, care must be taken to identify and avoid high-risk medications when able. This compendium serves to expedite the process of cross-referencing against a facility medication list, thereby saving time and effort in deciding on appropriate care for patients with AGS. For instance, volatile anesthetics are, across the board, safe for patients with AGS as they have no mammalian-derived ingredients, whereas clinicians should exercise increased caution with anticoagulant and antiplatelet medications as they are generally more likely to contain ingredients that can cause an AGS reaction. Furthermore, intravenous medications are generally safer than oral formulations as they require fewer fillers and inactive ingredients.

In summary, clinicians should be aware of AGS, as well as the most common triggers in the perioperative setting, and be prepared to treat if symptoms of a hypersensitivity reaction develop. With so many medications having the potential to contain alpha-gal and the exact content of alpha-gal in ingredients not readily quantifiable, the goal of this review is to serve as a reference and assist in providing safe care for our patients.

## References

[1] Dunkman WJ, Rycek W, Manning MW (2019). What does a red meat allergy have to do with anesthesia? Perioperative management of alpha-gal syndrome. Anesth Analg.

[2] Chung CH, Mirakhur B, Chan E (2008). Cetuximab-induced anaphylaxis and IgE specific for galactose-alpha-1,3-galactose. N Engl J Med.

[3] Fischer J, Yazdi AS, Biedermann T (2016). Clinical spectrum of α-Gal syndrome: from immediate-type to delayed immediate-type reactions to mammalian innards and meat. Allergo J Int.

[4] Wilson JM, Schuyler AJ, Schroeder N, Platts-Mills TA (2017). Galactose-α-1,3-galactose: atypical food allergen or model IgE hypersensitivity?. Curr Allergy Asthma Rep.

[5] Platts-Mills TA, Li RC, Keshavarz B, Smith AR, Wilson JM (2020). Diagnosis and management of patients with the α-Gal syndrome. J Allergy Clin Immunol Pract.

[6] Levin M, Apostolovic D, Biedermann T (2019). Galactose α-1,3-galactose phenotypes: lessons from various patient populations. Ann Allergy Asthma Immunol.

[7] Mullins RJ, James H, Platts-Mills TA, Commins S (2012). Relationship between red meat allergy and sensitization to gelatin and galactose-α-1,3-galactose. J Allergy Clin Immunol.

[8] Commins SP, James HR, Kelly LA (2011). The relevance of tick bites to the production of IgE antibodies to the mammalian oligosaccharide galactose-α-1,3-galactose. J Allergy Clin Immunol.

[9] Raghavan RK, Peterson AT, Cobos ME, Ganta R, Foley D (2019). Current and future distribution of the lone star tick, Amblyomma americanum (L.) (Acari: Ixodidae) in North America. PLoS One.

[10] Platts-Mills TA, Commins SP, Biedermann T (2020). On the cause and consequences of IgE to galactose-α-1,3-galactose: A report from the National Institute of Allergy and Infectious Diseases Workshop on Understanding IgE-Mediated Mammalian Meat Allergy. J Allergy Clin Immunol.

[11] Springer YP, Jarnevich CS, Barnett DT, Monaghan AJ, Eisen RJ (2015). Modeling the present and future geographic distribution of the lone star tick, Amblyomma americanum (Ixodida: Ixodidae), in the Continental United States. Am J Trop Med Hyg.

[12] Rutkowski K, Wagner A, Rutkowski R, Sowa P, Pancewicz S, Moniuszko-Malinowska A (2020). Alpha-gal syndrome: an emerging cause of food and drug allergy. Clin Exp Allergy.

[13] Rispens T, Derksen NI, Commins SP, Platts-Mills TA, Aalberse RC (2013). IgE production to α-gal is accompanied by elevated levels of specific IgG1 antibodies and low amounts of IgE to blood group B. PLoS One.

[14] Hamsten C, Starkhammar M, Tran TA (2013). Identification of galactose-α-1,3-galactose in the gastrointestinal tract of the tick Ixodes ricinus; possible relationship with red meat allergy. Allergy.

[15] Galili U (2013). Anti-Gal: an abundant human natural antibody of multiple pathogeneses and clinical benefits. Immunology.

[16] D’Ercole F, Dhandha V, Levi M, Todd A, Kumar P (2019). Perioperative challenges in patients with alpha-gal allergy. J Clin Anesth Pain Manag.

[17] Caponetto P, Fischer J, Biedermann T (2013). Gelatin-containing sweets can elicit anaphylaxis in a patient with sensitization to galactose-α-1,3-galactose. J Allergy Clin Immunol Pract.

[18] Holbert SE, Patel D, Rizk T, Dimitri NG, Jones M (2020). Intraoperative anaphylaxis in response to hemostatic agents with protein derivatives. Cureus.

[19] Commins SP (2020). Diagnosis & management of alpha-gal syndrome: lessons from 2,500 patients. Expert Rev Clin Immunol.

[20] Nourian MM, Stone CA Jr, Siegrist KK, Riess ML (2023). Perioperative implications of patients with alpha gal allergies. J Clin Anesth.

[21] Hawkins RB, Wilson JM, Mehaffey JH, Platts-Mills TA, Ailawadi G (2021). Safety of intravenous heparin for cardiac surgery in patients with alpha-gal syndrome. Ann Thorac Surg.

[22] Kim MS, Straesser MD, Keshavarz B, Workman L, McGowan EC, Platts-Mills TA, Wilson JM (2020). IgE to galactose-α-1,3-galactose wanes over time in patients who avoid tick bites. J Allergy Clin Immunol Pract.

[23] Commins SP, Jerath MR, Cox K, Erickson LD, Platts-Mills T (2016). Delayed anaphylaxis to alpha-gal, an oligosaccharide in mammalian meat. Allergol Int.

[24] Commins SP, James HR, Stevens W (2014). Delayed clinical and ex vivo response to mammalian meat in patients with IgE to galactose-alpha-1,3-galactose. J Allergy Clin Immunol.

[25] Fischer J, Lupberger E, Hebsaker J (2017). Prevalence of type I sensitization to alpha-gal in forest service employees and hunters. Allergy.

[26] Stone CA Jr, Hemler JA, Commins SP, Schuyler AJ, Phillips EJ, Peebles RS Jr, Fahrenholz JM (2017). Anaphylaxis after zoster vaccine: Implicating alpha-gal allergy as a possible mechanism. J Allergy Clin Immunol.

[27] (2023). National Library of Medicine. DailyMed. https://dailymed.nlm.nih.gov/dailymed/.

